# Factors associated with older people’s long-term care needs: a case study adopting the expanded version of the Anderson Model in China

**DOI:** 10.1186/s12877-017-0436-1

**Published:** 2017-01-31

**Authors:** Yuan Yuan Fu, Yu Guo, Xue Bai, Ernest Wing Tak Chui

**Affiliations:** 1 Department of Social Work and Social Administration, The University of Hong Kong, Room 712, Jockey Club Tower, The University of Hong Kong, Pokfulam, Hong Kong, China; 2 Department of Social Security, School of Labor and Human Resources, Renmin University of China, Room 227, Qiu Shi Building, No. 59 Zhongguancun Avenue, Beijing, 100872 China; 3 Department of Applied Social Sciences, The Hong Kong Polytechnic University, Hung Hom, Kowloon, Hong Kong, China; 4 Department of Social Work and Social Administration, The University of Hong Kong, 5/F Jockey Club Tower, The University of Hong Kong, Pokfulam, Hong Kong, China

**Keywords:** Long-term care needs, Older people, Anderson Model, Influencing factors, Psychosocial factors

## Abstract

**Background:**

Alongside changes in society and the economy, the family’s function of taking care of older people is weakening and the formal care mode is becoming more accepted. Older Chinese people are facing diverse choices of long-term care (LTC) modes. Acknowledging this situation, to optimize older people’s arrangements for LTC services and improve quality of later life, this study sets out to explore and make theoretical sense of older people’s LTC needs and to identify the factors influencing their LTC needs.

**Methods:**

Questionnaire data were collected from 1090 participants in four Chinese cities in 2014. A conceptual framework was established based on the Anderson Model (i.e., predisposing factors, enabling factors, and need factors), and further strengthened by adding several psychosocial factors (i.e. intergenerational relationships, unmet care service needs, and self-image). Multinomial logistic regression was adopted to explore the influencing factors of LTC needs. Participants choosing home-and-community-based care were regarded as the reference group.

**Results:**

After controlling for predisposing, enabling, and need factors, those with better self-image (*OR* = 1.027, *p* = 0.021) and fewer unmet care service needs (*OR* = 0.936, *p* = 0.009) were identified as being more likely to choose family care; those with less close intergenerational relationships (*OR* = 0.676, *p* = 0.019), fewer unmet care service needs (*OR* = 0.912, *p* = 0.027), and better self-image (*OR* = 1.044, *p* = 0.026) were more likely to choose institutional care. Gender- and age-related differences in the determinants of LTC needs were observed.

**Conclusions:**

The findings of this study suggest that professionals and service providers should pay more attention to the important role of psychosocial factors in affecting older people’s LTC needs and be more sensitive to gender- and age-related differences. Effective efforts to improve intergenerational relationships, to further develop care services for older people, and to foster a more positive image of aging should be emphasized.

## Background

The trend of population aging is increasingly becoming a major challenge for many advanced economies in the world. China has also entered the ranks of these aging countries according to the standard set by the World Health Organization. The rapid growth of the aging population has made the provision of sufficient long-term care (LTC) services for older people a critical social problem. In order to forecast the demand for LTC services and related costs, it is necessary to explore and make theoretical sense of older people’s LTC needs and to identify the factors that influence them [48].

Informal and formal LTC services coexist, complementing or substituting for each other [39]. Informal care is mostly delivered by family members. Although family care at home is the preferred choice in China, with the acceleration of urbanization, this kind of traditional care mode is facing increasing challenges due to the changes in caregivers’ motivation, resources, and time; the greater spatial distance between offspring and old people; and the changes in family structure [23, 38, 67].

On the other hand, formal care can be delivered either in the community in the form of home-and community-based care (HCBC), or in the institutional settings in the form of institutional care. To ease the caregiving burden on the younger generation, and to share the responsibility of elderly support between government and family members, several laws and regulations related to elderly care have been promulgated in China, including a new law on social insurance, and a proposed revision of the law on protecting the rights and benefits of older people [20]. In China, formal care is gaining ground [4, 18]. For institutional care, developed countries have already built up relatively advanced service delivery systems for older people’s institutional care, and there are also comprehensive government policies and regulations to monitor the quality of service delivery, for example, the White Paper “Caring for Our Future: Reforming Care and Support” in England, the Act on Prevention of Elderly Abuse and Support for Attendants of Elderly Persons in Japan, the Omnibus Budget Reconciliation Act of 1987 in the US. In China, institutional care is still in short supply compared with that in the developed countries. Nursing homes can provide beds for only 2.72% of the total number of older people [47]. For HCBC, although the lack of adequate and affordable institutional care and the weakening of traditional family care have made HCBC an appealing option, in China, HCBC is still at the early stages compared with that in developed countries. China is facing challenges regarding how to address mounting service needs with limited social resources in a young, developing civil society [68]. With the more recent developments in formal LTC provision, older Chinese people now have wider choices in LTC modes. In response to this situation, to optimize older people’s arrangements for LTC services and improve the quality of later life, this study sets out to explore and make theoretical sense of older people’s LTC needs, and to clarify influencing factors.

### Application of the Anderson model

To integrate influencing factors on older people’s LTC needs, the majority of researchers adopt a self-designed model [30, 32, 40, 52, 59, 61]; some also adopt the Anderson Model or its expanded version as theoretical model [11, 53, 62, 70]. The Anderson Model was initially designed in the late 1960s to explain or predict health service use [1]. Nowadays, it has been widely used in the study of older people’s actual use or intended use of services [11, 25, 28, 42, 66].

The initial phase of the Anderson Model includes several variables that can be categorized into three groups: predisposing characteristics, enabling factors, and need factors. A range of predictors that may explain older people’s LTC needs have been identified in previous studies, including: a) predisposing characteristics such as age, gender, educational level, and marital status, which are consistently found to be strong predictors [11, 40, 66, 70]; b) enabling factors, such as financial condition, number of children, and contact frequency with children are usually reported to be strong predictors [40, 42, 69]; c) need factors, activities of daily living (ADL), instrumental activities of daily living (IADL), and number of diseases usually show a significant impact [19, 24, 46, 65].

### Psychosocial factors and older people’s LTC needs

Parallel with the development of research on health service utilization and needs, the Anderson Model has evolved. In Bradley and colleagues’ opinion, although the Andersen Model includes “beliefs”, limited attention has been given to psychosocial factors: “These factors may be more important for long-term versus acute care because LTC involves assistance with routine personal tasks, about which individuals may have specific knowledge and strong attitudes” ([9], pp. 1223), which is consistent with the social psychology theory of planned behavior [9]. Bradley and colleagues’ expanded version of the Anderson Model focuses on intended use rather than actual use [9]. Considering their significance in particular cultural contexts, psychosocial factors have been included and tested when examining the factors influencing intended use of care services among older people and caregivers [15, 41].

In contemporary China, older people are a special cohort of people who have experienced a series of great changes in regard to culture, society, and economy, whose experiences are unique and distinct from older people in Chinese communities in other parts of the world. Since 1949, social policy in China has undergone changes in different periods, from socialism to modernization in the context of socio-economic and political development. Nowadays, older people are provided with more diverse choices of formal care and support services from the government and non-government sector, as a supplement to the original family care. Older people’s understanding of social norms, especially filial piety, has changed as a result of rapid cultural, social, and economic transformations [16, 33]. Older Chinese people’s knowledge of or attitudes toward LTC services may change as LTC services develop, and as changes occur in traditional culture (i.e., filial piety, familism, etc.), caregivers’ motivation, resources and time, and in family structure and pattern of living arrangements. Great changes have occurred not only in the development of LTC services, but also in older people’s psychosocial factors. To date, however, research examining the role of psychosocial factors in influencing older people’s LTC needs is limited. This study addresses gaps in testing the role of psychosocial factors in Chinese context.

According to Bradley and his colleagues’ expanded version of the Anderson Model, psychosocial factors included three domains in this model: (a) social norms; (b) attitude and knowledge concerning the use of long-term care services; (c) perceived control,which referred to individuals’ perceived ability to affect their choices for long-term care [9]. Referring to previous studies on psychosocial factors in studies of LTC needs [42, 58, 62, 64], intergenerational relationships, unmet care service needs, and self-image, which corresponds to social norms, attitude and knowledge and perceived control, were selected as older people’s psychosocial factors.

#### Intergenerational relationships and older people’s LTC needs

Intergenerational relationships seem to be a commonly used psychosocial factor by the researchers when they study on older people’s LTC needs in China [15, 62, 63]. A significant impact of intergenerational relationships on older people’s LTC needs has been found in some studies [15, 60, 62, 63, 77], while not in others [60, 77]. The impact of intergenerational relationships is still questionable. According to Stryker’s role-identity theory, along with the loss of work role in later life, older people’s family role identities and their relationships with their children likely become their main source of attachment and well-being [57]. In the Chinese context, taking care of older relatives is an obligation and traditionally a highly regarded practice [34]. Due to the rising standards of living, increasing educational levels, urbanization, and changes in children’s socialization, family structures and values are diversifying greatly [18, 31], which may result in strain and conflict in intergenerational relationships. In a culture that traditionally emphasizes the family’s role in caring for the elderly, influenced by the value of filial piety, the impact of these changes on LTC needs among older Chinese people may be even greater than in other nations. An examination of the potential impact of intergenerational relationships on older adults’ LTC needs has both theoretical and practical merits.

#### Unmet needs and older people’s LTC needs

Older people living at home, especially those with physical limitations, generally need support from others to perform daily life activities [51]. Unmet care service needs may have negative consequences for both older people and their family caregivers, such as increasing the utilization of health services, older people’s risk of health problems and sense of insecurity, and depressive symptomatology [2, 12, 14, 22, 37, 54, 58]. When older people’s care needs are not met, they may experience anxiety and a sense of insecurity, leading them to seek help from various sources, which may range from informal to formal care. However, studies on older people’s unmet care service needs in China are very limited. Given that a good understanding of older people’s unmet care service needs would offer an indicator of future care service needs [58], it is worth examining them and how much they affect older people’s LTC needs.

#### Self-image and older people’s LTC needs

Few attempts have been made to understand how older people in Chinese communities perceive themselves or how others perceive them [34, 76]. Evidence shows that older people residing in societies with a strong tradition of filial piety, like the Chinese, are experiencing a rapid decline in their self-image [5, 8]. As the value of respecting old people is quickly diminishing in importance in Chinese communities, the traditional image of aging is now in doubt [13, 17, 18]. It may be that negative self-image inclines older people to exert self-restraint and refrain from expressing their needs [64]. Whether the deterioration of older people’s self-image is further linked to their LTC needs has to be further explored.

### Gender- and age-related differences in the factors related to LTC needs

Previous studies have suggested gender as a predictor for LTC needs, but findings are inconsistent [11, 26, 44, 45]. Older women have been found to be much more likely than male counterparts to have lower socioeconomic status and to be financially dependent on others [71, 73]. Moreover, older women are much more likely to have lower levels of ADL, physical condition, cognition, and self-rated health [71]. The gender disparities on socioeconomic status and health may gradually translate into different experiences of aging and different factors influencing LTC needs [66].

Age-related disparities in the potential factors of LTC needs are frequently observed [7, 55]. Potential factors have been found to differ among different age groups [42, 62, 65]. To date, age-related differences in the influencing factors of LTC needs have yet to be empirically and fully explored [66]. Both researchers and practitioners should be more sensitive to such gender- and age-related differences.

In summary, investigation of the relationship between psychosocial factors and LTC needs is still in the early stages. Inspired by Bradley’s expanded version of the Anderson Model, this study is hoped to contribute to the understanding and exploration of the determinants of older people’s LTC needs in China and to test the role of psychosocial factors. Additionally, older people of different gender and age may take different factors into account when expressing their LTC needs [66]. Therefore, this study was also designed to explore gender- and age-related differences in the influencing factors of LTC needs among older Chinese people.

## Methods

### Sampling

Data were collected from January to October 2014 in four cities (Beijing, Guizhou, Heibi, and Xi’an). Participants in each city were selected using multistage stratified sampling. The sampling process involved a systematic approach and a four-step scheme: (a) A sampling frame was set based on the administration districts in each city. (b) Each district was then divided into census blocks and a list of elders living in each of the selected blocks was created. (c) By population proportional sampling, the specified number of samples to recruit was set. (d) Investigators have resorted to random sampling method to recruit older people in each block with the help of street-level officials. The screening criteria included: a) being older than 60, b) having at least one child, and c) having normal cognitive functions. Five undergraduate students and seven graduated students from Renmin University of China were recruited and trained as interviewers. They were clearly informed of the purposes of the study. Before the interview, all the participants were given a brief introduction about the purpose of this study, and asked to sign in the consent form. Each interview lasted for about 40 min. 1118 participants were interviewed. The response rate was 76.6%. For data cleaning, missing values represented 3% or more of observations for any given variable were excluded. Ultimately, 1090 samples were analyzed in this study.

### Measures

In this study, older people’s LTC needs were regarded as the dependent variable, measured by a single question: “Which mode of LTC would you like to choose?” (possible responses: 1 = family care, 2 = HCBC, 3 = institutional care). Following the expanded version of the Anderson Model, four groups of independent variables were assessed in this study: predisposing characteristics, enabling factors, need factors, and psychosocial factors.

#### Predisposing characteristics

The variables included in predisposing characteristics were: age (1 = 60-69, 0 = 70 or above), gender (0 = female, 1 = male), educational level (1 = primary school or below, 2 = junior or senior high school, 3 = college or above), and marital status (0 = currently unmarried, 1 = currently married). To control the diverse characteristics across cities, “region” was also included in this study (1 = Beijing, 2 = Guizhou, 3 = Hebi, 4 = Xi’an).

#### Enabling factors

Information was collected on a number of enabling factors: income, number of children, and contact frequency with children. Personal annual income and number of children were measured by continuous variables. Contact frequency with children was measured by a single question “How frequently do you contact with your children?”. Responses were rated from 1 (infrequently) to 3 (frequently).

#### Need factors

Two variables were included in need factors: IADL and number of diseases. IADL was measured with eight items by the Lawton Instrumental Activities of Daily Living Scale [35]. The IADL was thus scored out of 8; a higher score implying greater independence in daily life. The scale had good internal consistency with a Cronbach’s alpha (α) of 0.85. Number of diseases was measured by the multiple-choice question, “How many chronic diseases do you have?” Nine chronic diseases were listed for selection, including cardiovascular and cerebrovascular diseases, hypertension, arthropathy, rheumatism, diabetes, respiratory disease, cataract,digestive diseases and others; a higher score meant that the participant had more chronic diseases.

#### Psychosocial factors

Three variables were added to measure psychosocial factors: intergenerational relationships, unmet care service needs and self-image. A scale with three questions was used to measure intergenerational relationships: “How are your relationships with your children?” “How do you get along with your children?” and “Do you communicate well with your children?” Responses were rated from 1 (very bad or very little) to 6 (very good or very often). The average score for these three questions was regarded as indicating the quality of intergenerational relationships on a scale of 1 to 6; a higher score indicated closer intergenerational relationships. The scale had good internal consistency with a Cronbach’s alpha (α) of 0.91. Older people’s unmet care service needs were measured on four aspects of HCBC services: daily care (five types of services), living environment (two types of services), medical treatment (four types of services), and spiritual life (two types of services). To measure unmet care service needs, older people were asked two dichotomous questions: “Is this type of service available to you?” (0 = unavailable, 1 = available), and “Do you need this type of service?” (0 = need, 1 = not need). For those who expressed themselves but did not receive the service, their unmet care service needs were coded 1. For those who expressed no need, and those who expressed that their needs had been satisfied, their unmet care service needs were coded 0. The maximum score for unmet needs was 13; a higher score meant a higher level of unmet needs. Self-image was measured by the Chinese version of the Self-Image of Aging Scale (SIAS-C) [3]. The 14-item SIAS-C was used to investigate how older people saw themselves in five aspects: general physical health, social virtues, life attitudes, psychosocial status, and cognition. The scores on this scale ranged from 14 to 70; a higher score meant a more positive self-image. The internal consistency of SIAS-C in this study was 0.81 (Cronbach’s alpha).

### Data analysis

Chi-square tests were used to test the relationships between potential factors and older people’s LTC needs. As the most appropriate statistical model for categorical dependent variables, multinomial logistic regression was used to explore the influencing factors. The expanded version of the Anderson Model was incorporated into the logistical model by adding predisposing characteristics, enabling factors, need factors, and psychosocial factors. To separately examine the variance explained by variables representing different groups in the Anderson Model and to test the role of psychosocial factors on older people’s LTC needs after controlling the first three groups of variables (i.e., predisposing characteristics, enabling factors, and need factors), stratified models were adopted. Moreover, multinomial logistic regressions of LTC needs stratified by gender and age were conducted separately to further investigate the differences of influencing factors among different groups (between male and female, younger group and older group). The statistical Package for Social Sciences (SPSS) version 21.0 (Chicago, IL, USA) was used for data analysis.

## Results

### Profile of participants

Table 1 shows the characteristics of the samples and the differences among older people choosing family care, HCBC, and institutional care. Among the 1090 participants, 75.3% (*N* = 821) chose family care, 16.7% (*N* = 182) chose HCBC, and 8.0% (*N* = 87) chose institutional care; 51.5% (*N* = 561) were aged 60 to 69, 48.5% (*N* = 529) were aged 70 or above; 47.5% (*N* = 518) were women; 52.5% (*N* = 572) were men. Over half of the sample (53%) had a high school education (*N* = 578). Participants with educational level of primary school or below and college or above represented 19.9% (*N* = 217) and 27.1% (*N* = 295) respectively. Less than a fifth, 17.2% (*N* = 187), were unmarried at the time of the study. There were 414, 232, 236, and 208 participants from Beijing, Guizhou, Heibi, and Xi’an respectively. The mean value of personal annual income was RMB35,838 (*SD* = 23,931). The average number of children was about two; 3.6% (*N* = 39) had infrequent contact with children, 11.5% had occasional contact, and 85% (*N* = 926) had frequent contact with children. The mean score of IADL was 7.324, and the mean number of diseases was about one. The mean score for intergenerational relationships was 4.707 out of 6. The mean number of unmet care service needs was about five. The mean score of self-image was 54.599 out of 70. Through chi-square tests, significant differences among participants choosing different LTC modes were found on educational level (*p* = 0.020), region (*p* = 0.001), number of children (*p* = 0.001), contact frequency with children (*p* < 0.001), number of diseases (*p* = 0.041), intergenerational relationships (*p* < 0.001), unmet care service needs (*p* = 0.001), and self-image (*p* = 0.023).

**Table 1:** Differences in characteristics among participants choosing family care, HCBC and institutional care mode

	Total (*N* = 1090)		Family care (*N* = 821)		HCBC (*N* = 182)		Institutional care (*N* = 87)		p
	*N*	%	*N*	%	*N*	%	*N*	%	
Predisposing characteristics									
Age									0.211
60-69	561	51.5	410	49.9	102	56.0	49	56.3	
70 or above	529	48.5	411	50.1	80	44.0	38	43.7	
Gender									0.219
Female	518	47.5	378	46.0	96	52.7	44	50.6	
(Male)	572	52.5	443	54.0	86	47.3	43	49.4	
Educational level									0.020
Primary school or below	217	19.9	170	20.7	37	20.3	10	11.5	
Junior high school or senior high school	578	53.0	444	54.1	89	48.9	45	51.7	
(College or above)	295	27.1	207	25.2	56	30.8	32	36.8	
Marital status									0.198
Currently not married	187	17.2	136	16.6	30	16.5	21	24.1	
(Currently married)	903	82.8	685	83.4	152	83.5	66	75.9	
Region									0.001
BJ	414	38.0	298	36.3	69	37.9	47	54.0	
GZ	232	21.3	165	20.1	62	34.1	5	5.7	
HB	236	21.7	180	21.9	32	17.6	24	27.6	
(XA)	208	19.1	178	21.7	19	10.4	11	12.6	
Enabling factors									
Income (thousand, RMB) (mean, SD)	35.838, 23.931		35.622, 24.710		36.787, 22.971		35.701, 17.789		0.050
Number of children (mean, SD)	2.220, 1.176		2.297, 1.182		2.002, 1.142		1.954, 1.109		0.001
Contact frequency with children									<0.001
Infrequently	39	3.6	21	2.6	8	4.4	10	11.5	
Sometimes	125	11.5	91	11.1	19	10.4	15	17.2	
(Frequently)	926	85.0	709	86.4	155	85.2	62	71.3	
Need factors									
IADL (mean, SD)	7.324, 1.585		7.354, 1.536		7.233, 1775		7.233, 1.631		0.561
Number of diseases (mean, SD)	1.179, 1.003		1.136, 0.992		1.303, 1.036		1.333, 1.008		0.041
Psychosocial factors									
Intergenerational relationships (mean, SD)	4.707, 0.855		4.755, 0.801		4.654, 0.882		4.364, 1.164		<0.001
Unmet care service needs (mean, SD)	4.581, 3.590		4.450, 3.579		5.423, 3.693		4.058, 3.225		0.001
Self-image (mean, SD)	54.599, 8.591		54.922, 8.456		53.007, 8.467		54.880, 9.771		0.023

### Factors associated with older people’s LTC needs

Table 2 shows the relative risk ratios from the full model of the multinomial logistic regression of LTC needs, including predisposing characteristics, enabling factors, need factors and psychosocial factors. HCBC was used as the reference. Participants from Beijing, Guizhou, and Hebi (compared with Xi’an: Beijing, *OR* = 0.474, *p* = 0.009; Guizhou, *OR* = 0.265, *p* < 0.001; Hebi, *OR* = 0.382, *p* = 0.005) and those with more unmet care service needs (*OR* = 0.936, *p* = 0.009) were less likely to choose family care. Participants with more children (*OR* = 1.268, *p* = 0.011) and with more positive self-image (*OR* = 1.027, *p* = 0.021) were more likely to choose family care. Participants in Guizhou (compared with Xi’an: *OR* = 0.138, *p* = 0.001) and with a lower score on intergenerational relationships (*OR* = 0.676, *p* = 0.019) and more unmet care service needs (*OR* = 0.912, *p* = 0.027) were less likely to choose institutional care. Participants who were currently unmarried (*OR* = 2.362, *p* = 0.016) and had a more positive self-image (*OR* = 1.044, *p* = 0.026) were more likely to choose institutional care. Table 3 shows the changes in model fit across the four regression models. Four multinomial logistic regression models were estimated for LTC needs. Change in model fit was calculated. Predisposing factors had the greatest explanatory power on the observed difference in LTC needs (Pseudo *R* ^2^ = 0.082). The addition of enabling factors yielded a 2.7% improvement of explanatory power (Chi-square = 93.912, *p* < 0.001). The addition of need factors yielded a minor improvement of 1.2% (Chi-square = 104.799, *p* < 0.001). The addition of psychosocial factors yielded an improvement of 2.7% (Chi-square = 130.070, *p* < 0.001). The full regression model accounted for 14.8% of the total variance of LTC needs.

**Table 2:** Multinomial logit coefficients of older people’s LTC needs

	Family care vs. HCBC		Institutional care vs. HCBC	
	*p*	OR	*p*	OR
Predisposing characteristics				
Age				
70 or above	0.724	1.075	0.763	0.908
(60–69)				
Gender				
Female	0.121	0.761	0.995	1.002
(Male)				
Educational level				
Primary school or below	0.142	1.504	0.153	0.492
Junior high school or senior high school	0.075	1.438	0.962	0.985
(College or above)				
Marital status				
Currently not married	0.765	1.074	0.016	2.362
(Currently married)				
Region				
BJ	0.009	0.474	0.626	1.241
GZ	0.000	0.265	0.001	0.138
HB	0.005	0.382	0.919	0.949
(XA)				
Enabling factors				
Income	0.458	0.974	0.479	0.952
Number of children	0.011	1.268	0.628	1.077
Contact frequency with children				
Infrequently	0.405	0.681	0.115	2.453
Sometimes	0.262	1.384	0.058	2.180
(Frequently)				
Need factors				
IADL	0.366	1.055	0.380	0.922
Number of diseases	0.154	0.883	0.784	1.038
Psychosocial factors				
Intergenerational relationships	0.682	1.046	0.019	0.676
Unmet care service needs	0.009	0.936	0.027	0.912
Self-image	0.021	1.027	0.026	1.044

**Table 3:** Changes in model fits for older people’s LTC needs

	Regression models	Pseudo R^2^	Change	Chi-square
1	Predisposing	0.082		69.908^***^
2	Predisposing + enabling	0.109	0.027	93.912^***^
3	Predisposing + enabling + need	0.121	0.012	104.799^***^
4	Predisposing + enabling + need + psychosocial	0.148	0.027	130.070^***^

^***^ *p* < 0.001

### Gender differences on influencing factors of LTC needs

To determine the gender differences in influencing factors of LTC needs, this study conducted two more regression models that regressed LTC needs in key independent variables for males and females respectively (Table 4). Region, number of children, and self-image were three factors significantly related to males’ preference of family care, while region and unmet care service needs were two significant factors for females. Region, contact frequency with children, intergenerational relationships, and self-image were four potential factors affecting male participants’ preference for institutional care, while marital status, region, and unmet care service needs were three significant factors for female participants.

**Table 4:** Multiple logistic regression of LTC needs stratified by gender

	Male				Female			
	Family care vs. HCBC		Institutional care vs. HCBC		Family care vs. HCBC		Institutional care vs. HCBC	
	*p*	OR	*p*	OR	*p*	OR	*p*	OR
Predisposing characteristics								
Age								
70 or above	0.338	1.323	0.753	1.159	0.469	0.810	0.382	0.667
(60–69)								
Educational level								
Primary school or below	0.512	1.312	0.532	0.624	0.094	1.952	0.176	0.379
Junior high school or senior high school	0.062	1.686	0.445	1.409	0.425	1.283	0.419	0.690
(College or above)								
Marital status								
Currently not married	0.300	0.679	0.618	1.341	0.114	1.666	0.004	3.984
(Currently married)								
Region								
BJ	0.041	0.374	0.901	0.915	0.116	0.557	0.436	1.592
GZ	0.001	0.196	0.036	0.145	0.004	0.324	0.015	0.110
HB	0.016	0.278	0.874	0.881	0.145	0.489	0.972	1.026
(XA)								
Enabling factors								
Income	0.192	0.949	0.984	1.001	0.525	1.043	0.286	0.873
Number of children	0.009	1.464	0.263	1.291	0.209	1.170	0.728	0.926
Contact frequency with children								
Infrequently	0.991	1.008	0.019	6.739	0.378	0.579	0.307	0.279
Sometimes	0.729	1.148	0.340	1.780	0.172	1.833	0.071	2.909
(Frequently)								
Need factors								
IADL	0.731	0.967	0.145	0.818	0.147	1.122	0.746	1.045
Number of diseases	0.506	0.915	0.615	1.112	0.298	0.882	0.731	1.068
Psychosocial factors								
Intergenerational relationships	0.535	1.107	0.019	0.569	0.976	0.996	0.342	0.790
Unmet care service needs	0.342	0.964	0.798	0.984	0.004	0.904	0.013	0.867
Self-image	0.029	1.039	0.019	1.070	0.326	1.016	0.456	1.020

### Age differences on influencing factors of LTC needs

To determine the age differences in influencing factors of LTC needs, two regression models for participants aged 60–69 (younger group) and 70 or above (older group) were conducted (Table 5). Educational level, region, and unmet care service needs were three factors significantly related to the younger group’s preference for family care, while region, number of children, and self-image were three significant factors for the older group. Marital status, region, intergenerational relationships, unmet care service needs, and self-image were five potential factors affecting younger participants’ preference for institutional care, while the contact frequency with children was the significant factor for the older group.

**Table 5:** Multiple logistic regression of LTC needs stratified by age

	60-69				70 or above			
	Family care vs. HCBC		Institutional care vs. HCBC		Family care vs. HCBC		Institutional care vs. HCBC	
	*p*	OR	*p*	OR	*p*	OR	*p*	OR
Predisposing characteristics								
Gender								
Female	0.784	0.937	0.664	1.184	0.053	0.591	0.743	0.862
(Male)								
Educational level								
Primary school or below	0.030	2.360	0.071	0.202	0.817	0.905	0.588	0.668
Junior high school or senior high school	0.003	2.263	0.736	1.148	0.543	0.814	0.735	0.832
(College or above)								
Marital status								
Currently not married	0.211	1.676	0.005	4.965	0.714	0.892	0.281	1.708
(Currently married)								
Region								
BJ	0.010	0.230	0.338	0.482	0.177	0.623	0.260	1.951
GZ	0.000	0.104	0.005	0.090	0.013	0.377	---	---
HB	0.002	0.151	0.082	0.232	0.590	0.768	0.064	4.085
(XA)								
Enabling factors								
Income	0.668	0.979	0.354	0.902	0.635	0.972	0.751	1.034
Number of children	0.156	1.242	0.465	0.819	0.007	1.403	0.198	1.291
Contact frequency with children								
Infrequently	0.051	0.333	0.862	0.878	0.418	2.472	0.024	16.675
Sometimes	0.818	0.919	0.651	0.765	0.108	2.296	0.002	7.701
(Frequently)								
Need factors								
IADL	0.474	0.895	0.962	0.987	0.665	1.031	0.479	0.925
Number of diseases	0.095	0.797	0.166	1.337	0.400	0.902	0.371	0.826
Psychosocial factors								
Intergenerational relationships	0.812	1.036	0.024	0.600	0.910	1.019	0.411	0.787
Unmet care service needs	0.027	0.921	0.012	0.858	0.140	0.949	0.579	0.966
Self-image	0.440	1.012	0.032	1.059	0.028	1.040	0.408	1.025

## Discussion

Using the expanded version of the Anderson Model, this study explores older people’s LTC needs in China. Significant differences in educational level, marital status, region, number of children, and contact frequency with children were examined. The role of psychosocial factors (intergenerational relationships, unmet care service needs, and self-image) in affecting older people’s LTC needs was emphasized. The differences by gender and age in influencing factors of LTC needs were also confirmed.

With respect to the distribution of older people’s preference of LTC mode, in the four cities, the majority of older people still preferred the concept of “aging in place” and receiving family care (*N* = 821, 75.3%) or HCBC services (*N* = 182, 16.7%); only 8.0% chose institutional care (*N* = 87). Older people choosing family care were still a large majority. As the core of Confucianism, familism provides a strong tie among generations. Intergenerational reciprocity, especially filial piety, is one of the behavioral principles establishing children’s caregiving duty to their parents. The ethics of caring reflects the principle of reciprocity between generations. China’s government usually keeps its hands off the family due to the emphasis on family throughout China’s history [21, 75]. This explains why HCBC services are not yet well developed in China. A considerable percentage of the older participants selected formal care, which indicates the importance of both HCBC and institutional care in the LTC system. HCBC services always have been regarded as an extension of family caregiving rather than a replacement for it [36]. Results showed that older people had greater acceptance to HCBC than institutional care. It is recommended that all relevant stakeholders prioritize HCBC development and improve the quality and quantity of HCBC services to satisfy the needs of older people who prefer to engage formal care services in the community. The government should encourage the provision of more support for older people and take action to improve formal care services.

In the determinants of LTC needs among all the samples, predisposing characteristics (marital status and region), enabling factors (number of children and contact frequency with children), and psychosocial factors (intergenerational relationships, unmet care service needs, and self-image) were found in this study. For predisposing characteristics, the findings of the significant effect of marital status are supported by many studies [56, 66, 72, 77]. The findings of regional disparity may be caused by differences in geographical location, city size, level of economic development, level of LTC development, and cultural contexts among the four cities sampled. For enabling factors, the findings were consistent with those of other studies [42, 62, 72]. No significant relationships were found between the two need factors (IADL and number of diseases) and LTC needs, which contradicts the findings of some studies [10, 66, 77]. This may be caused by the limited number of IADLs and diseases of the samples: the mean IADL score was 7.324 out of 8, which revealed that the majority of the participants were healthy. The small difference in need factors among samples may be the cause of the non-significant relationship with LTC needs.

The role of psychosocial factors on older people’s LTC needs is well documented in this study: 2.7% of the variance was explained by variables representing psychosocial factors after controlling the other three groups of variables. This confirmed the important role of psychosocial factors in affecting LTC needs, which is corresponding to the results of previous studies [15, 41]. The significant correlation between psychosocial factors and LTC needs indicates that, first, older people with closer relationships with their children prefer the concept of “aging in place” no matter whether they are receiving informal care services (family care) or formal care services (HCBC). In fact, even HCBC services must be supplemented by viable care by family members. Thus, solidarity between older people and their children, a virtue in Chinese culture, is a crucial factor enabling older people to continue to live in their familiar domestic environment, thus achieving “aging in place”.

Second, compared to those choosing family care and institutional care, older people choosing HCBC were more likely to express their unmet care service needs. On the one hand, the limited availability of community care resources restricts the satisfaction of older people’s LTC needs, thus leaving their needs unmet. On the other hand, older people choosing HCBC are more inclined to express their needs of care services offered by the community than those choosing family care or institutional care. Previous studies reveal that unmet care service needs may lead to negative consequences for both older people and their caregivers. However, there are few studies on older people’s unmet care service needs in China. These unmet care service needs, which explain the theoretical link between needs and utilization of LTC, deserve more attention in future study.

Third, in this study, self-image consisted of five factors: general physical health, attitude toward life, cognition, social virtues, and psychosocial status [3]. People with a positive self-perception of their general physical health, life attitude, psychosocial status, and cognition may think they are well able to take care of themselves, being actively engaged in an activity or with society, and thus would like to stay at home and receive family care. Two social virtues, filial piety and social face, are important cultural values in China. Therefore, older Chinese people care about how society or other people perceive them, and especially about whether others show them the respect they are due [27]. To avoid the loss of social face, many older Chinese people try their best to show their better side and to cover up their less desirable aspects [29]. Staying at home and receiving help from their offspring is a way of enjoying filial support and also a way of saving face. Contrarily, it would be a disgrace if older people could not be cared for by their adult children [43]. That is why, as revealed by this study, older Chinese people with better self-image prefer to stay at home and receive care support from their family rather than engage HCBC services.

Another interesting point is that for the participants choosing formal care (institutional care or HCBC), those preferring institutional care had better self-image than those preferring HCBC. On the one hand, this may be related to the underdevelopment of the institutional care system in China. Unlike developed countries, China has implemented a residual social welfare system in which the family plays the crucial role of social support system. Older people who receive support from their family are usually unable to obtain the welfare and relief directly provided by the government. The high cost and strict eligibility rules mean that the majority of older people in institutions are those with greater financial resources or higher positions before retirement. Those who are in higher social classes may have a more positive self-image. On the other hand, several studies indicate that Chinese people’s understanding of filial piety may have changed [16, 33, 50]. Providing financial assistance should be regarded as a part of being filial. The increasing unavailability of adult children, various benefits, and the high cost of institutional care are contributing to a shift in attitudes about institutional care, from stigma to privilege [74]. Therefore, in cases where older people lack informal caregiving support, those who can afford the high cost of institutional care with financial support from children may have a more positive self-image than those who receive formal care services in the community.

To further explore differences in influencing factors of older people’s LTC needs, we conducted regressions by gender and age groups were conducted. With respect to gender, the results reveal that there are different factors affecting LTC needs of older men and women. Referring to the life course perspective and the feminist perspective [6], the socially constructed roles of men and women, and the gender-based division of labor in economic and family spheres in earlier life stages may gradually translate into different experiences of aging, which helps to explain the gender differences in the influencing factors of older people’s LTC needs.

With respect to age, the influencing factors of the 60–69 and 70 or above age groups are totally different. This could be attributed to the fact that in China, where life expectancy is about 74.83 [49], older people reaching 70 years old may perceive the imminence of LTC needs while people under 70 perceive such needs as being more remote.

## Conclusions

There are very few studies examining the role of psychosocial factors in influencing factors of older people’s LTC needs and testing gender- and age-related differences in this issue in China. This study has provided useful insights into the determinants of older people’s LTC needs theoretically and practically. Theoretically, it could be postulated that subjective factors may be equally, if not more, important in affecting older people’s expression of their LTC needs. Specifically, cultural factors may subtly affect older people’s values and attitudes, which in turn influence their behavior. Therefore, we suggest further investigation of the effect of psychosocial factors on LTC needs. This study, though based on findings in the Chinese context, could provide a reference point for other societies, especially those that similarly emphasize familial relationships and caregiving. Gender- and age-related differences in the determinants of LTC needs have been determined, and should be emphasized. It is recommended that researchers and service providers be more sensitive to these differences when studying or providing LTC.

In practice, although family care is still the preferred choice, formal care, especially HCBC, is becoming increasingly accepted, a development which deserves more attention from the Chinese government. Nowadays, the development of HCBC has become a growing industry in China with great support by the Chinese government’s policies, such as Views on Promoting the Work of Home Care for Older People in 2008, Policy Directives to Promote Home Care in 2009, Overall Policy Direction for the Development of Services for Older People and so on. More efforts should be made to enhance older people’s utilization of LTC services in the community. This study makes a humble contribution to enriching the Anderson Model by highlighting some psychosocial factors, which adds to our understanding of LTC needs. The present findings suggest that intergenerational relationships, unmet care service needs, and older people’s self-image have significant relationships with their LTC needs. Effective efforts to enhance older people’s preference to HCBC should focus on improving intergenerational relationships, further developing care services for older people, and paying more attention to those with lower positive image of aging. When working with older men to enhance their HCBC needs, social workers should help to raise their contact frequency with children, improve intergenerational relationships and pay more attention to those with less children and with lower positive image of aging. When working with older women to enhance their HCBC needs, further developing care services is more effective, and more attention should be paid to unmarried older women. When working with younger group of older people, all the three domains of psychosocial factors work to enhance their preference to HCBC. Moreover, social workers should pay more attention to those with lower educational level and unmarried ones. Publicity efforts to change their mind (i.e. introducing more on HCBC) are necessary to improve their receptivity to HCBC. While, when working with older group of older people to enhance their preference to HCBC, social workers should pay more attention to those with less children and with lower positive self-image, help to raise the contact frequency between older people and their children.

We should note that there are several limitations in this study. First, its cross-sectional nature impedes our ability to make cause-and-effect conclusions. The impact of predisposing characteristics, enabling factors, need factors, and psychosocial factors cannot be determined and still need further evidence. Second, this study only recruited community-dwelling older people in urban areas. Studies of older people in institutions and in rural areas should be conducted in the future. Third, considering that a limited number of IADLs and diseases were found among the samples, studies on the generalizability of the findings to frail older people needs further exploration.

## Abbreviations

ADL: Activities of daily living
HCBC: Home-and community-based care
IADL: Instrumental activities of daily living
LTC: Long-term care
SIAS-C: The Chinese version of the self-image of aging scale
